# Efficient Ultrasound-Assisted Extraction of Four Major Aescins from *Aesculi Semen* Seeds Using Deep Eutectic Solvents

**DOI:** 10.3390/molecules31061057

**Published:** 2026-03-23

**Authors:** Su Bu, Jia Yang, Qifeng Xu, Hui Sun, Xiyu Yang, Xunyong Zhou, Linguo Zhao, Xuhui Zhang

**Affiliations:** 1 College of Life Science, Nanjing Forestry University, Nanjing 210037, China; jyang95@njfu.edu.cn (J.Y.);; 2 College of Ecology and Environment, Nanjing Forestry University, Nanjing 210037, China; 3 HC Enzyme (Shenzhen) Biotech. Co., Ltd., Shenzhen 518112, China; 4 College of Chemical Engineering, Nanjing Forestry University, Nanjing 210037, China; 5 College of Forestry, Nanjing Forestry University, Nanjing 210037, China

**Keywords:** *Aesculi Semen*, aescin, deep eutectic solvent, green extraction

## Abstract

Low extraction efficiency limits the availability and application of aescins, which exhibit various pharmacological activities. Here, we optimized parameters for ultrasound-assisted extraction of aescins from *Aesculus chinensis* seeds using deep eutectic solvent (DES)-water mixtures. Seven DES formulations were screened, and one providing the highest yield was selected for optimizing the molar ratio. The effects of four parameters were investigated using single-factor experiments combined with response surface methodology. The optimal extraction conditions were as follows: DES, a 1:1 mixture of 1,3-butanediol and lactic acid, with 42.5% water, used at a liquid-solid ratio of 25 mL/g; ultrasonic frequency, 40 kHz; extraction temperature, 70 °C; and extraction time, 27.5 min. The extraction yield under these conditions was significantly higher than that obtained via traditional methods. Aescin was purified from the DES extract using macroporous resin. AB-8 resin was most efficient in adsorbing aescin in static adsorption tests. Based on dynamic adsorption experiments, optimal separation, with a 100% recovery rate, was achieved by passing four bed volume (BV) of extract through AB-8 column, removing impurities with two BV of deionized water and four BV of 30% ethanol, and eluting with four BV of 60% ethanol at 5–10 mL/min. This green method should be suitable for large-scale applications.

## 1. Introduction

Dried seeds of *Aesculus chinensis* Bge. or *Aesculus wilsonii* Rehd. from the Hippocastanaceae family, commonly referred to as Suoluozi, are used as a traditional Chinese medicine [1]. Suoluozi is rich in various bioactive components, including flavonoids, coumarins, sterols, organic acids, amino acids, and triterpenoid saponins, of which flavonoids, coumarins, and triterpenoid saponins have attracted considerable attention of researchers owing to their unique biological activities [2]. Aescin, a collective term used for a mixture of more than 30 triterpenoid saponins extracted from horse chestnut seeds, exhibits multiple pharmacological effects, including anti-inflammatory [3,4], antitumor [5,6,7,8], neuroprotective [9], gastroprotective [10], analgesic [11], antimicrobial [12], and antiviral properties [13,14]. It has also been reported to regulate energy metabolism and improve vascular tone [15,16]. Clinically, it is used to treat thrombotic diseases such as cerebral thrombosis [17,18], regulate intestinal dysfunction [19], and manage various inflammatory conditions [20]. Notably, aescin demonstrates corticosteroid-like anti-inflammatory and circulation-improving effects [21] with no hormonal side effects [22] or contraindications [23]. These properties make aescin a pharmacologically versatile agent with broad therapeutic potential.

**Figure 1:**
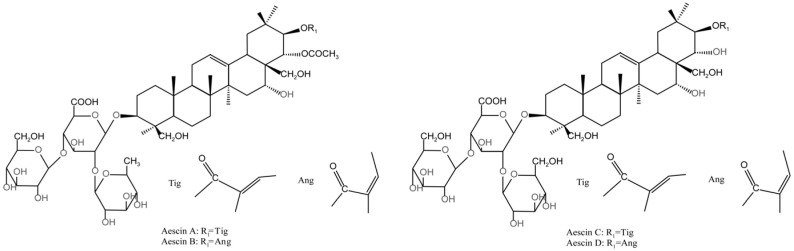
Chemical structures of aescin A, aescin B, aescin C, and aescin D [29].

Aescin comprises four main components: aescin Ia (aescin A), aescin Ib (aescin B), isoescin Ia (aescin C), and isoescin Ib (aescin D) [24]. Aescin A and B, also called β-aescin [25], are the two most abundant types [26], whereas aescin C and D, referred to as α-aescin, rank second [27,28]. These four aescin components (Figure 1) account for over 60% of the total aescin content [29]. Aescin A, B, C, and D are isomers, differing only in the structure of the terminal terpene ring—the COCH_2_ group is attached to the OH group in aescin A and B, whereas the COCH_2_ group is linked to the CH_2_OH group in aescin C and D [30].

Deep eutectic solvents (DESs) are solvents formed by mixing hydrogen bond donors (HBDs) and hydrogen bond acceptors (HBAs) at specific molar ratios through intermolecular hydrogen bonding [31]. Because of their low cost, biodegradability, biocompatibility, and ease of preparation, DESs are considered promising green alternatives to traditional solvents [32]. Typical HBAs include quaternary ammonium salts, whereas common HBDs include polyols, organic acids, and sugars. The combination of solid raw materials forms a eutectic mixture, wherein hydrogen bonds or van der Waals forces disrupt the crystalline structure of the original solids, resulting in a liquid state, with excellent solvent properties, at ambient temperature [33]. In recent years, DESs have been widely used for extracting high-value bioactive compounds [34]. DESs are generally nontoxic; their components are safe and environment friendly, and some DESs can even be prepared using food-grade chemicals. The application of DES significantly improves extraction efficiency while preserving the bioactivity of the extracts. Moreover, DESs are nonvolatile, and can therefore reduce the extraction time. Johnatt et al. [35] demonstrated the potential of DES in extracting flavonoids from citrus pomace by combining pectinase-assisted DES extraction of phenolic compounds.

Aescin is readily soluble in alcohols, particularly methanol [29]. Industrially, aescin is predominantly extracted using ethanol or continuous reflux extraction [27,28]. For research purposes, methanol-based ultrasonic extraction is the most commonly employed method for aescin [29].

The use of DES for aescin extraction from seeds of (*Aesculus chinensis* Bge has not been reported. In this study, we explored the application of green DES as extraction solvents, in conjunction with stable, efficient, and low-hazard ultrasound-assisted extraction (UAE). Multiple DES systems were screened, and the most efficient DES was selected for parameter optimization using single-factor experiments and central composite design (CCD) response surface methodology (RSM). Furthermore, we employed AB-8 macroporous resin to purify the four major aescin components from the crude DES extract.

Ultrasound-assisted extraction (UAE) is recognized as a green and efficient technique that enhances the recovery of bioactive compounds from plant materials [36]. The mechanism of UAE relies on acoustic cavitation, where the formation and violent collapse of microbubbles in the solvent generates high-speed micro-jets and shear forces. These forces physically disrupt plant cell walls, facilitating solvent penetration into the cellular matrix and accelerating the mass transfer of intracellular compounds into the extraction solvent [37,38]. Studies on triterpenoid saponins from various medicinal plants have shown that parameters such as ultrasonic power, temperature, and extraction time critically influence extraction efficiency, and optimization of these parameters using RSM can substantially enhance saponin recovery [39].

## 2. Materials and Methods

### 2.1. Experimental Materials and Instruments

Crushed, dried, and sieved (30–40 mesh) *Aesculus chinensis* seed powder was supplied by Jiangsu Gushen Biotechnology Co., Ltd. (Yanchen, China). Reference standards of aescin A, B, C, and D (98% purity) and aescin/sodium aescinate (97% purity) were obtained from Yuanye Bio-Technology (Shanghai, China). Components for DES (HBDs/HBAs) were purchased from Aladdin Reagent and Titan Scientific (Shanghai, China). Macroporous resin was sourced from Solarbio and Mitsubishi Chemical (Tokyo, Japan). Chromatographic acetonitrile and methanol were acquired from Tedia Company (Fairfield, OH, USA). Other reagents used in the study included acetic acid (Titan Scientific), sulfuric acid (Nanjing Reagent, Nanjing, China), ferric chloride (Aladdin), phosphoric acid (Nanjing Reagent), ethanol (Wuxi Yasheng Chemical Co., Ltd., Wuxi, China), and chromatographic methanol (Tedia).

The sources of equipment were as follows: temperature-controlled stirring water bath (JY-4), Changzhou Tianjing Laboratory Instrument Factory, Changzhou, China; ultrasonic water bath (HY-200DH), Shanghai Yuhao Company, Shanghai, China; constant-temperature shaker (MQD-S2R), Shanghai Minquan Instrument Company, Shanghai, China; centrifuge (5910R, Eppendorf, Hamburg Germany); iMark microplate reader, Bio-Rad Laboratories (Hercules, CA, USA); ACQUITY Arc-2998 ultra-performance liquid chromatography system, Waters Corporation (Milford, MA, USA).

### 2.2. High-Performance Liquid Chromatography (HPLC) and Standard Curve Preparation

The four primary aescin monomers (A, B, C, and D) were quantified via HPLC, adapting a detection method from the Chinese Pharmacopoeia (2015 Edition) with optimized mobile phase parameters. The chromatographic conditions were: mobile phase, a 36:64 mixture of acetonitrile:water (0.2% phosphoric acid); flow rate, 1.0 mL/min; detection wavelength, 220 nm; column temperature, 25 °C; injection volume, 10 μL.

The four aescin reference standards (aescin A, B, C, and D) were individually dissolved and diluted with methanol to prepare 10 mg/mL stock solutions. These stocks were mixed to create gradient standard solutions containing 1, 0.5, 0.4, 0.3, 0.2, 0.1, and 0.05 mg/mL of each aescin component. Standard curves were plotted with mass concentrations on the x-axis and peak areas on the y-axis (Table S1).

### 2.3. DES Preparation and Screning

Considering the low melting point of DESs [40,41], we adopted a heating method for preparation [42]. Based on DESs reported for compounds structurally similar to the four aescins, seven DESs (Table 1) capable of forming stable transparent liquids under mild preparation conditions (≤80 °C, ≤2 h) were selected as potential solvents for initial extraction. Preliminary extraction of aescins was conducted using these seven DESs under initial extraction conditions (30% water content, 20:1 liquid:solid ratio, 60 °C, 30-min ultrasonic extraction). The aescin content was determined via HPLC, and the DES with the highest extraction yield was selected for further optimization. For comparison, control groups were established based on optimal conditions reported in the literature for ethanol reflux extraction and methanol ultrasonic-assisted extraction [43,44].

### 2.4. Optimization of DES Extraction Conditions and RSM Design

Based on the results of preliminary screening, DES#1, with the highest extraction yield, was selected for single-factor experiments to examine the effects of different extraction parameters on the efficiency of aescin extraction. Initially maintaining the screening conditions for DES#1 (30% water content, 1:20 solid:liquid ratio, 60 °C, 30 min ultrasonic extraction), the effects of four key factors, including, water content, temperature, extraction time, and liquid-solid ratio, on aescin extraction yield (mg/g) was assessed and optimized using RSM with Design Expert version 12.0.3.

Because single-factor experiments have limitations, we used the relatively optimized parameters while considering the cost and safety requirements of actual production in factory (i.e., avoiding excessively high temperatures (<80) and excessively high solid:liquid ratios (<25) to design a four-factor, five-level RSM (Table 2), and the extraction yield of the four aescins was used as a response value. The interactive effects of the independent variables were assessed using a CCD, comprising a total of 30 experimental points including 6 center runs to ensure the repeatability and stability of the extraction process. Extraction conditions were screened based on the extraction yield.

### 2.5. Adsorption and Recovery of Four Aescins Using Macroporous Resin

Macroporous resin enables effective separation and elution of target substances with similar characteristics by adjusting the polarity of the eluent, thereby rendering efficient recovery and enrichment of the target compounds. To ensure its optimal performance, macroporous resin was pretreated according to the manufacturer’s instructions. Briefly, the resin was soaked for 4 h in ethanol, with concentrations exceeding 90%. The ethanol was filtered off, and the resin was washed with deionized water until completely free of alcoholic odor. Finally, the resin was suction-filtered to dryness, weighed, and stored for later use.

For the dynamic adsorption experiment, the macroporous resin was packed into a glass chromatographic column using a wet method with 95% ethanol. The column was gently tapped to eliminate air bubbles and then kept soaked for 4 h. After draining the ethanol, deionized water was slowly added along the inner wall of the column, and the valve was opened to release the water while maintaining the liquid level above the resin bed to prevent exposure to air. This step was repeated several times until the effluent was free of alcoholic odor. Following this pretreatment, a uniform layer of quartz sand (thickness, approximately 0.5 cm) was placed on top of the resin bed to stabilize it and prevent floating during subsequent operations. Finally, the macroporous resin column was pre-equilibrated with a solution matching the DES concentration of the sample to be loaded.

#### 2.5.1. Selection of Macroporous Resin Using Static Adsorption

To enrich and purify aescins from the crude DES extract, we screened resins with different polarities through static adsorption and desorption tests. The resin with the highest adsorption efficiency was selected for dynamic adsorption experiments.

Five types of macroporous resins, D101, ADS-17, AB-8, NKA-9, and polyamide, corresponding to nonpolar, neutral, weak-polar, polar, and strong-polar characteristics, were selected. Pretreated resins were weighed and mixed with the crude DES extract of aescin at a mass:volume ratio of 1:4. The mixtures were horizontally shaken at 150 rpm for 6 h at room temperature and then filtered through a filter paper to obtain the post-adsorption effluent. The concentrations of aescins in the initial sample solution and effluent were measured. The adsorption rate was calculated using Equation (1) to select the resin with the highest adsorption rate for subsequent dynamic experiments.

The adsorption capacity (A) and adsorption rate (B%, percentage) were calculated as follows:

A = (ρ_0_ − ρ_1_) V_1_/m (1) B% = (ρ_0_ − ρ_1_)/ρ_0_ × 100%

where A is the adsorption capacity (mg/g), B% is the adsorption rate (%), ρ_0_ is the initial mass concentration of target substance in the extract (g/L), ρ_1_ is the residual mass concentration of target substance in the adsorption solution (g/L), V_1_ is the volume of the sample solution (mL), and m is the mass of the resin (g).

Optimization of eluent concentration: Adsorption-saturated resin was vacuum-filtered and sequentially eluted with excess volumes of ethanol at four concentrations (50%, 60%, 70%, and 80%). The concentration of aescin eluted at different ethanol concentrations was measured. The optimal ethanol concentration in eluent for desorption was then determined and used in dynamic adsorption experiments.

#### 2.5.2. Optimization of Dynamic Adsorption

We further optimized key parameters in the adsorption and desorption processes in dynamic experiments; these included water content in the DES extract, adsorption time, ratio of loading extract volume to column BV, ethanol concentration in the eluent, ratio of eluent volume to column BV, and desorption time. These optimizations established fundamental conditions for dynamic desorption.

Optimization of sample loading: An excess volume of the aescin extract was loaded onto the column packed with the optimal resin. The flow rate was maintained at 10 mL/min, and the effluent was collected every 10 mL to monitor the leakage point for macroporous resin. This volume was used as the maximum sample loading volume for subsequent experiments.

Optimization of eluent concentration and volume: Based on the static adsorption experiment, four ethanol concentrations (50%, 60%, 70%, and 80%) were used for sequential gradient elution with excess volume. The eluate was collected per column bed volume (BV), and the concentrations of the four aescins were measured. The ethanol concentration yielding the highest elution efficiency was selected as the optimal desorption solvent. Subsequently, excess volume of this single-concentration ethanol was applied to determine the required eluent volume.

### 2.6. Reuse of DES and Macroporous Resins

To evaluate the reuse potential of DES, the post-adsorption DES effluent (after macroporous resin treatment) was reused for extracting aescin from *Aesculus chinensis* seeds. This DES recycling test was repeated for three extraction cycles, each with three replicates.

For regenerating the macroporous resin, the used resin column was first washed with 80–90% ethanol to remove low-polarity impurities, followed by rinsing with 3–4 BV of deionized water until the effluent became ethanol-free. The column was then re-equilibrated with fresh DES (identical to the initial loading solution) before subsequent sample loading and elution steps.

### 2.7. Statistical Analysis

The experimental data were analyzed and processed using Excel 2019 and SPSS 26 software. All the data represent the mean values from three independent biological replicates. A threshold of *p* < 0.05 was set to determine statistical significance.

## 3. Results

### 3.1. HPLC Standard Curve

**Figure 2:**
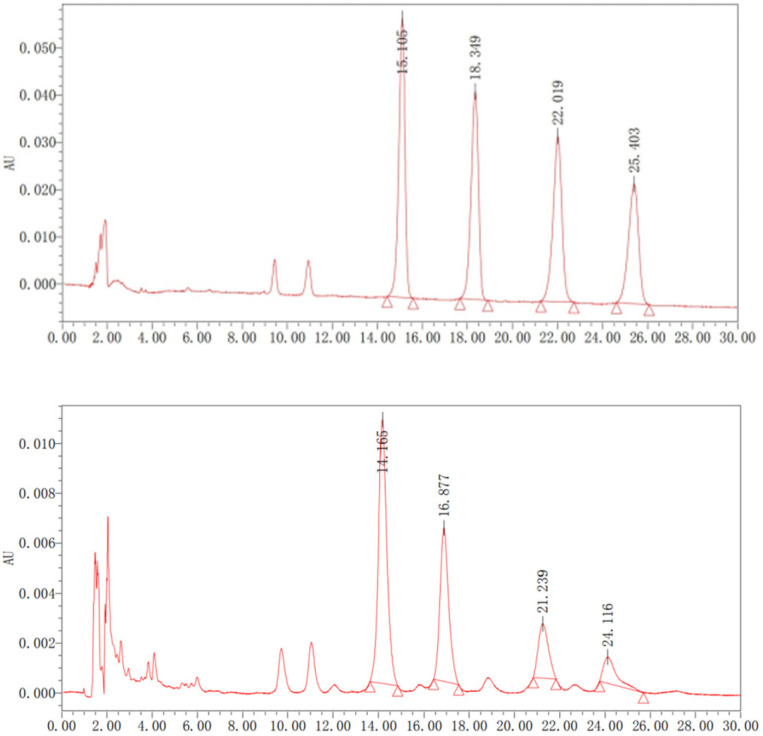
HPLC profiles of mixed reference standards of the four aescins (aescin A, B, C, and D, >99%) (**top**) and the crude extract of aescins from DES (**Bottom**).

The mixture of four aescins (>98%) was analyzed using the HPLC method. The regression equations for aescin A, B, C, and D are presented in Table 2. The chromatographic peaks of reference standards are shown in Figure 2.

### 3.2. DES Screening

#### 3.2.1. Comparison of Extraction Efficiency Achieved with DES and Traditional Alcohol Extraction Methods

**Figure 3:**
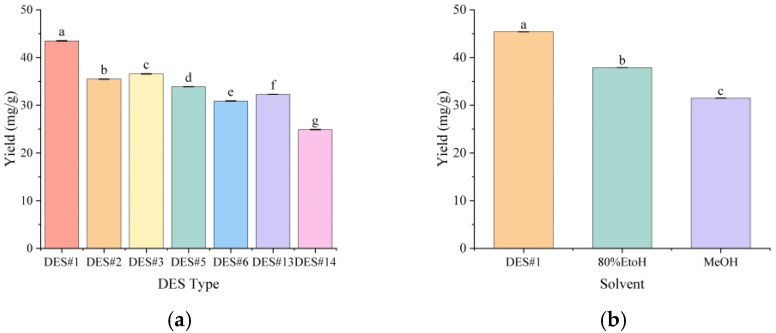
Initial screening results with ethanol reflux extraction and methanol ultrasonic extraction: (**a**) Result of preliminary deep eutectic solvent (DES) screening; (**b**) Comparison of DES#1, ethanol reflux extraction, and methanol ultrasonic extraction. Different letters indicate statistically significant differences between groups (*p* < 0.05).

The DES#1 formulation (1,3-butanediol:Lactic acid = 1:1 molar ratio), which demonstrated the highest extraction efficiency, was selected for subsequent experiments. Even under unoptimized moderate conditions, DES#1 achieved an extraction yield of 43.5 mg/g (Figure 3a), significantly outperforming conventional methanol (31.55 mg/g) and ethanol (37.93 mg/g) extraction methods as reported in the literature (Figure 3b).

#### 3.2.2. Optimization of the Molar Ratio of HBA and HBD in DES#1

**Figure 4:**
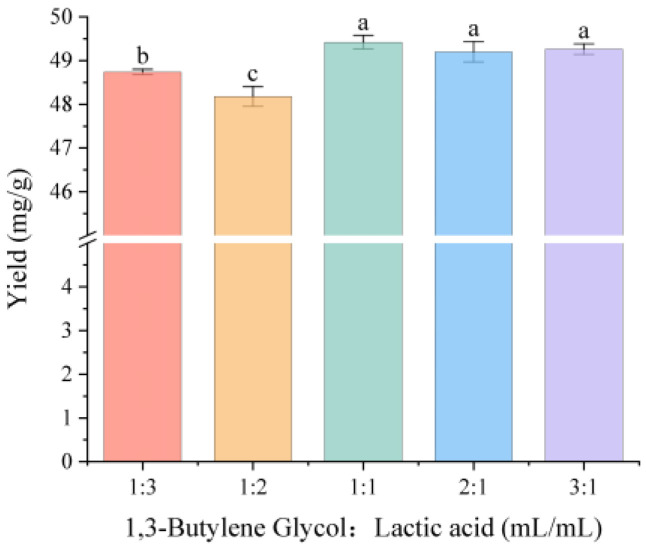
Effect of the molar ratio of hydrogen bond acceptor and hydrogen bond donor in DES#1 on the extraction yield. Different letters indicate statistically significant differences between the groups.

Based on the extraction yields, we further optimized the molar ratio of DES#1 to investigate potential improvements in the extraction yield of the four aescins. As shown in Figure 4, variations in the molar ratio did not significantly affect the yield. Therefore, subsequent experiments were performed using DES prepared at the original 1:1 molar ratio.

### 3.3. Single Factor Experiments

**Figure 5:**
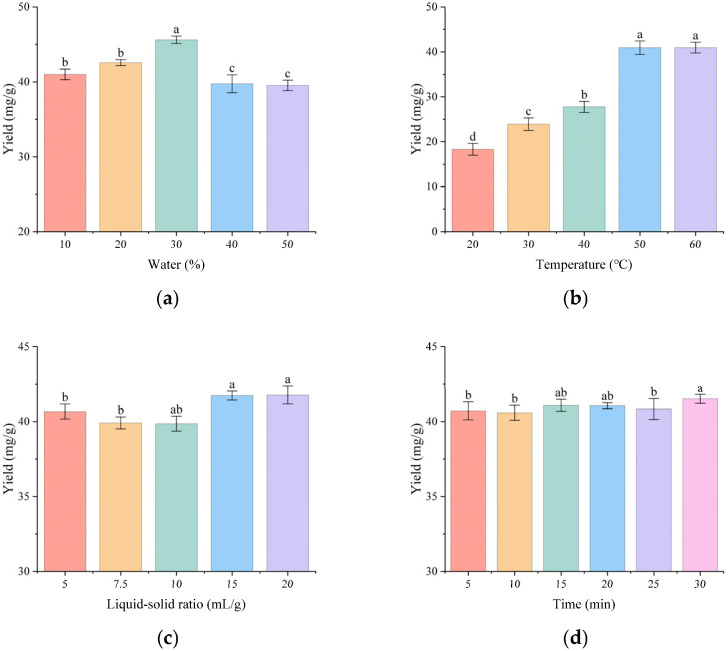
Effect of single extraction condition factors on the extraction rate: (**a**) Time; (**b**) Water content; (**c**) Temperature; (**d**) Liquid:solid ratio. Different letters indicate statistically significant differences between the groups.

We first conducted single-factor experiments to evaluate key extraction parameters—water content, extraction temperature, liquid:solid ratio, and extraction time. Optimal extraction efficiency was obtained at 30% water content (Figure 5a) and 50 °C extraction temperature (Figure 5b), whereas variations in the liquid:solid ratio (Figure 5c) and extraction time (Figure 5d) had minor effect on the yield.

### 3.4. Response Surface Analysis for Optimizing Extraction Conditions

As single-factor experiments cannot fully reflect the interactions between variables or help determine optimal conditions, a four-factor CCD experiment using Design Expert 12.0.3 (Table 3) was employed. The experimental outcomes centered on extraction yield (Y), quantified as milligrams of the sum of four aescins per gram of *Aesculi Semen* seeds. Extraction results for a total of 30 experimental points are presented in Table 3. The yield of four major aescins ranged from 38.42 mg/g to 85.76 mg/g.

After performing multivariate nonlinear regression fitting of the experimental data, the quadratic polynomial regression equations for the extraction yield of aescin (Y_1_) were determined to establish predictive models, enabling the determination of empirical relationships between the independent variables and extraction yield of aescin (Y_1_), as exemplified below.

Y_1_ = 62.72 + 1.45 A + 1.23 B + 11.82 C + 0.4557 D − 0.0351 AB + 0.0242 AC − 0.1427 AD + 0.0076 BC − 0.2519 BD + 0.0534 CD − 0.5957 A^2^ − 0.1866 B^2^ − 0.0494 C^2^ − 0.2224 D^2^

Based on the quadratic regression model of the RSM and subsequent analysis, analysis of variance (ANOVA) was performed for the extraction yield, the results of which are presented in Table 4.

The RSM for extraction yield (Table 4) showed extremely high significance (*F* = 432.32, *p* < 0.0001) with a nonsignificant lack-of-fit term (*F* = 3.31, *p* = 0.0995), demonstrating excellent model fit and predictive accuracy while confirming minimal influence from uncontrolled variables, thus validating the reliability of the model for analyzing the effects of the four factors on the extraction yield of aescin.

The *F* and *p* values presented in Table 4 indicate complex effects of linear, quadratic, and interaction terms on the response variable, revealing neither simple linear nor quadratic relationships. Based on the *F* values, the factors influencing aescin extraction yield followed this order of significance: liquid:solid ratio > DES water content > extraction temperature > extraction time.

#### Analysis of Response Surface Results

**Figure 6:**
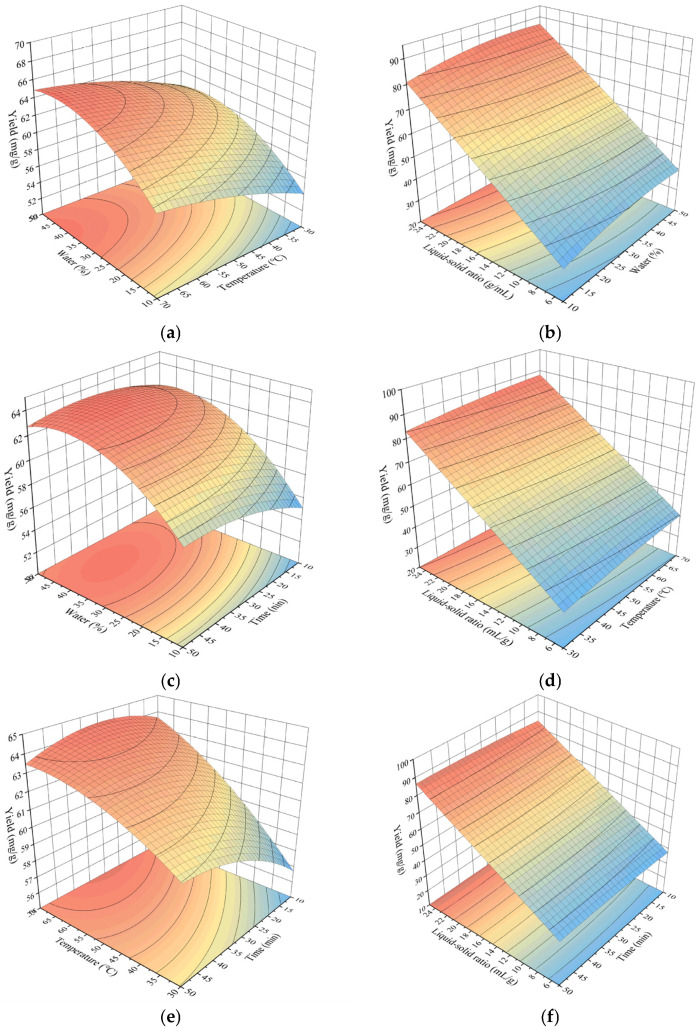
Three-dimensional response surface plots. Interaction of (**a**) temperature and water content; (**b**) liquid:solid ratio and water content; (**c**) time and water content; (**d**) liquid:solid ratio and temperature; (**e**) time and temperature; (**f**) time and liquid:solid ratio.

The effects of interactions between factors on the response surface model can be visualized through the three-dimensional response surface plots and contour maps presented in Figure 6.

The steeper the response surface, the more significant the interactive effects are. As evident from the steepness of the response surfaces in Figure 6b,d,f, the interactive effects of the liquid:solid ratio with the water content, extraction temperature, and extraction time were the most significant. In contrast, the flatter response surfaces in Figure 6a,c,e indicated that the interactive effects between the water content and time or temperature were less pronounced. However, Figure 6a,c show a slightly steeper response surface than Figure 6e, suggesting that the interactive effects between the water content and extraction temperature or extraction time were slightly stronger than those between temperature and time. This further validated the ANOVA results, with the order of influence being BD > AD > CD > AB > AC > BC.

### 3.5. Optimization According to CCD Results and Verification

Using the CCD RSM, the final predicted optimal extraction parameters were determined to be a water content of 42.364%, an extraction temperature of 70 °C, a liquid:solid ratio of 25:1, and an extraction time of 27.26 min, with a predicted extraction yield of 88.775 mg/g. For practical application, these parameters were rounded to a water content of 42.5%, temperature of 70 °C, liquid:solid ratio of 25:1, and extraction time of 27.5 min. Three independent experiments under these conditions yielded an average extraction yield of 87.947 ± 0.541 mg/g, which was significantly higher than the best result obtained after single-factor optimization (43.5 mg/g). The experimental values showed no significant difference from the predicted values, confirming that the established model is accurate, well-fitted, and objectively reliable. Compared with single-factor experiments, the CCD RSM achieved a much higher extraction yield. This further highlighted the superiority of the RSM optimization approach.

### 3.6. Separation and Recovery of Aescin Using Macroporous Resin

#### 3.6.1. Selection of Macroporous Resins with Different Polarities Using Static Adsorption

**Figure 7:**
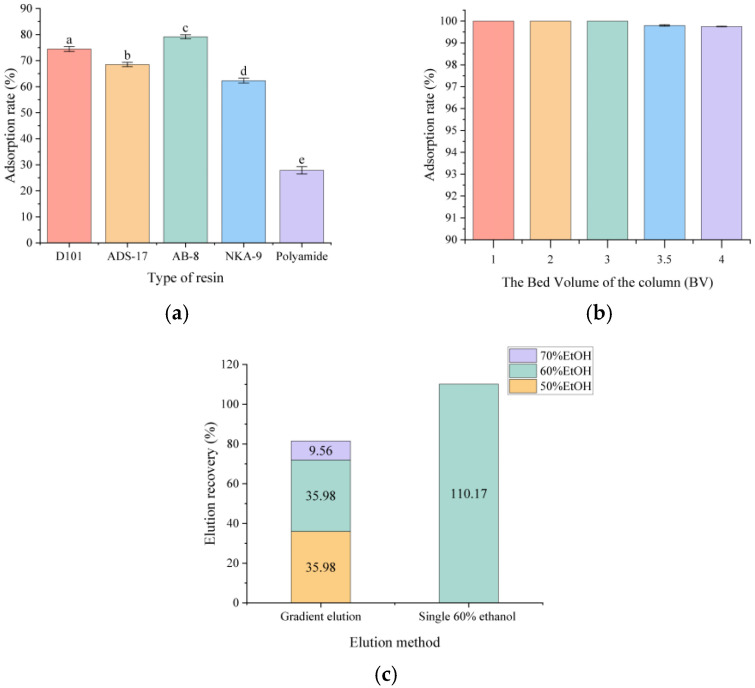
Adsorption rate of different resins in static adsorption (**a**); Adsorption rate of AB-8 macroporous resin with different bed volumes (BV) of loading solution in static adsorption (**b**); Recovery rate of aescin with gradient and single elution in dynamic desorption (**c**). In (**a**), the letters a–e indicate statistically significant differences between groups (p < 0.05).

Although static adsorption exhibits significantly lower efficiency in both adsorption and desorption compared with dynamic adsorption, it serves as an efficient preliminary screening method for resin selection by allowing rapid evaluation with minimal material and time investment through the assessment of recovery rate. We performed static adsorption using a four-fold resin BV of crude DES extract, followed by a standardized washing procedure: resins were placed on a MQD-S2R shaker (150 rpm, room temperature) and sequentially treated with four BV of water for 30 min (to remove residues), 30% ethanol for 2 h (to eliminate low-polarity impurities), and 60% ethanol for 2 h (for aescin recovery). Among the five resins, AB-8 macroporous resin showed the highest absorption capacity, achieving up to 80% recovery (Figure 7a). This resin was selected for subsequent optimization of dynamic adsorption and desorption parameters.

During the dynamic adsorption experiment, resin saturation (also called the breakthrough/leaking point) was determined by deliberately over-loading the DES crude extract onto column. An AB-8 resin column (42 cm high, 2.8 cm internal diameter) was run at 10 mL min^−1^, and effluent fractions were collected every 0.5 BV. HPLC analysis revealed that breakthrough occurred after loading four BV of the extract, indicating that the maximum adsorption capacity of the resin was three BV of the loading extract (Figure 7b). After adsorption, the column was washed with two BV of deionized water for removing residual solutes, followed by four BV of 30% ethanol for eluting the impurities. Gradient elution was carried out with two BV of 50%, 60%, and 70% ethanol, achieving aescin recovery rates of 35.98%, 35.98%, and 9.56%, respectively. An additional five column BV elution with 60% ethanol was performed in parallel. A single, continuous elution with five BV of 60% ethanol gave the highest recovery of 100%, whereas only ~80% recovery was achieved with the gradient elution scheme (Figure 7b).

#### 3.6.2. Multiple Utilization of AB-8 Resin and DES

Both the AB-8 resin and DES demonstrated excellent reusability. The AB-8 resin maintained 100% adsorption capacity even after four usage cycles, although the aescin recovery rate gradually declined to approximately 89%, indicating the need for resin regeneration after four uses (Table 5). Notably, while complete elution initially required five BV of 60% ethanol, this volume decreased to only three BV after several rounds of resin use, suggesting a reduction in the adsorption capacity of the resin. This phenomenon, coupled with the declining recovery rate, implied accumulation of irreversible adsorption (“dead adsorption”) on the resin surface, necessitating regeneration [45,46].

The DES extract system exhibited outstanding reusability performance with minimal efficiency loss in extraction yield across multiple cycles (Table 6). The slightly higher extraction yields observed in the second cycle may be attributable to residual aescins in the recycled DES. These findings confirm the feasibility of reusing both materials for industrial applications, offering significant benefits for cost reduction and environmental sustainability.

## 4. Discussion

In this study, efficient extraction of the four major aescins (aescin A, B, C, and D) from *A. chinensis* was achieved using ultrasound-assisted DES-water solution. These four components are isomers differing in the structure of the terminal terpene ring. The optimized DES#1 system demonstrated excellent extraction efficiency for all four monomers without preferential extraction, maintaining the natural compositional profile of the crude drug.

Due to the amphiphilic nature of aescin, the addition of water to the DES can enhance its hydrophilicity, alter the balance between some HBAs and HBDs within the DES, and lead to the formation of new HBA and HBD configurations. The superior performance of DES#1 (1,3-butanediol:lactic acid, 1:1 molar ratio) was attributed to the synergistic HBA/HBD interactions between its components. Lactic acid serves as both a hydrogen bond donor (HBD) through its carboxyl and hydroxyl groups and a mild acidifying agent. Its carboxyl group forms strong hydrogen bonds with the multiple hydroxyl and carboxyl moieties of aescins, while its acidic environment helps maintain the carboxyl groups of aescins in their protonated state (-COOH), enhancing solubility in the organic DES phase and preventing precipitation as poorly soluble salts. 1,3-Butanediol functions as a hydrogen bond acceptor in DES, contributing lipophilic character that facilitates dissolution of the hydrophobic triterpene backbone of aescins while participating in the hydrogen bond network with lactic acid. The equimolar combination creates an extensive, dense hydrogen bond network that disrupts plant cell wall architecture, competes with aescins’ hydrogen bonds to cell wall components, and incorporates the target compounds into the DES supramolecular structure through non-covalent interactions [47]. This aligns with the binding theory, emphasizes the importance of intermolecular interactions between HBA, HBD, and target molecules—which incorporate the solute into the supramolecular structure of the DES [48].

The optimization of extraction parameters provided insights into their mechanistic roles. Water content (optimized at 42.5%) critically influences extraction efficiency by reducing DES viscosity, enhancing mass transfer, and modulating the balance between HBA and HBD components to favor aescin solubilization. Due to the amphiphilic nature of aescins, water addition alters the hydrogen bond network and may create new configurations that facilitate solute incorporation. Temperature (optimized at 70 °C) enhances extraction through reduced viscosity, increased solubility, and improved diffusion coefficients, while remaining safely below the 100 °C threshold where β-aescins convert to α-aescins [49]. Higher solvent-to-material ratios increase the concentration gradient driving force for mass transfer and ensure complete wetting of plant material. The liquid-to-solid ratio (optimized at 25:1) showed the most significant influence (F-value = 5871.12, *p* < 0.0001), reflecting its role in maintaining concentration gradients and ensuring complete material wetting. However, excessively high ratios (>25:1) would lead to unnecessary solvent consumption without proportional yield improvement, while ratios below 15:1 resulted in incomplete extraction due to saturation effects and limited contact efficiency.

Extraction time (optimized at 27.5 min) demonstrates the efficiency of UAE, High-frequency sound waves induce cavitation bubble formation and violent collapse within the solvent, disrupting plant cell walls and facilitating rapid solvent penetration into the cellular matrix [36]. The relatively short optimal time (27.5 min) demonstrates the efficiency of UAE-DES combination. Prolonged extraction (>40 min) showed no yield improvement and potentially risks compound degradation or isomerization.

Compared with conventional methanol and ethanol extraction methods, which typically require longer extraction times, repeated extraction times and higher solvent consumption [44], Our UAE-DES approach not only operates at lower temperatures but also benefits from the nonvolatile nature of DES, ensuring both process stability and environment friendliness.

Wang et al. extracted aescin from Suoluozi powder with ethanol, followed by sequential extraction with ethyl acetate and *n*-butanol. The *n*-butanol extract was vacuum-dried, concentrated, and purified using NKA-9 resin and a reversed-phase chromatography column (Sep-Pak C18). The final product yield was 36.424 mg/g, with a recovery rate of 3.67% [43]. Gao et al. used reflux extraction with ethanol to obtain aescin from Suoluozi, with a yield of 23.9 mg/g [44]. However, in our study, several DESs achieved an aescin extraction yield exceeding 40 mg/g even without any optimization of key factors in preliminary experiments. Under the optimized conditions, an excellent extraction yield of 87.95 mg/g was obtained, significantly higher than those in the abovementioned studies.

AB-8 macroporous resin was identified as the most effective among five tested resins (D101, ADS-17, AB-8, NKA-9, and polyamide). This selection is supported by similar research, in which nine macroporous resins were systematically evaluated for purifying triterpenoid saponins, AB-8 was identified as exhibiting the highest adsorption capacity and desorption rate among all tested resins [47]. The weak-polar nature of AB-8 provides balanced affinity for the amphiphilic aescin molecules—sufficient to adsorb them from the polar DES-water extract while allowing efficient desorption with 60% ethanol. The adsorption mechanism involves hydrophobic interactions between the resin matrix and the triterpene aglycone moiety, complemented by hydrogen bonding between residual hydroxyl groups on the resin and the sugar chains of aescins. This dual interaction enables selective adsorption while excluding more polar impurities (removed by water and 30% ethanol washes) and less polar contaminants (retained during 60% ethanol elution). The complete recovery (100%) achieved with four BV of 60% ethanol confirms the suitability of AB-8 for industrial-scale purification of aescins from DES extracts.

UAE is an efficient, eco-friendly method for isolating bioactive compounds from plant materials. During the UAE process, high-frequency sound waves induce the formation of cavitation bubbles within the solvent. The rapid creation and violent collapse of these bubbles disrupt plant cell walls, thereby facilitating more efficient solvent penetration into the cellular matrix [36]. 

When designing single-factor experiments, we considered the practical conditions of safety and economy in industrial production, such as avoiding the used of excessively high extraction temperatures, excessive time, or excessive liquid-solid ratio. The optimal parameters obtained from the single-factor experiments were used as a reference, and optimization was carried out using RSM. We also attempted to reduce extraction costs by reusing the DES. The extraction efficiency of aescin was found to be the same after three cycles of DES reuse. However, the recovery rate decreased by approximately 10% after the AB-8 resin was reused four times. While these results may vary in large-scale production, they indicate that the DES-water extraction solvent can be recycled in a manner similar to ethanol, with additional advantages such as low volatility.

## 5. Conclusions

In this study, DES#1 (1,3-butanediol:lactic acid, 1:1) was selected from seven deep eutectic solvents for its superior extraction efficiency of four major aescins from Aesculus chinensis seeds. Using single-factor experiments and response surface methodology, the optimal extraction conditions were determined: 42.5% water content, 70 °C, 25 mL/g liquid-to-solid ratio, and 27.5 min, yielding 87.95 mg/g of aescins. For purification, AB-8 macroporous resin showed the highest adsorption capacity. Dynamic tests revealed that loading four bed volumes (BV) of crude extract achieved complete adsorption, while impurities were removed with two BV of water and four BV of 30% ethanol. The adsorbed aescins were fully recovered using four BV of 60% ethanol. This study establishes a green, efficient, and scalable DES-based ultrasound-assisted extraction and purification method for aescins.

## Figures and Tables

**Table 1:** Composition of deep eutectic solvents (DESs) used in this study.

DESs	HBA	HBD	Molar Ratio
DES#1	1,3-Butanediol	Lactic acid	1:1
DES#2	Betaine	Lactic acid	1:1
DES#3	*n*-Propanol	Acetamide	2:1
DES#4	*n*-Propanol	Lactic acid	1:1
DES#5	Choline chloride	Ethylene glycol	1:2
DES#5	Choline chloride	Lactic acid	1:2
DES#7	Choline chloride	Glycerol	1:2

**Table 2:** Design for the response surface methodology.

Factors	Level				
	−2	−1	0	1	2
Water (%)	10	20	30	40	50
Temperature (°C)	30	40	50	60	70
Liquid:solid ratio (mL/g)	5	10	15	20	25
Time (min)	10	20	30	40	50

**Table 3:** Central composite design and results for total aescin yield.

Run	Factors				Target Index
	A (%)	B (°C)	C (mL/g)	D (min)	Extraction Yield (mg/g)
1	20	40	10	20	47.134
2	40	40	10	20	50.134
3	20	60	10	20	49.798
4	40	60	10	20	52.277
5	20	40	20	20	70.000
6	40	40	20	20	73.831
7	20	60	20	20	73.724
8	40	60	20	20	75.838
9	20	40	10	40	48.459
10	40	40	10	40	50.344
11	20	60	10	40	49.867
12	40	60	10	40	52.591
13	20	40	20	40	72.404
14	40	40	20	40	74.251
15	20	60	20	40	73.412
16	40	60	20	40	76.096
17	10	50	15	30	56.376
18	50	50	15	30	63.436
19	30	30	15	30	58.409
20	30	70	15	30	64.675
21	30	50	5	30	38.419
22	30	50	25	30	85.762
23	30	50	15	10	59.836
24	30	50	15	50	62.961
25	30	50	15	30	62.159
26	30	50	15	30	62.392
27	30	50	15	30	63.021
28	30	50	15	30	63.094
29	30	50	15	30	62.360
30	30	50	15	30	63.306

A: water content, percentage; B: Extraction Temperature; C: Liquid-solid ratio; D: Extraction Time.

**Table 4:** Extraction yield response surface regression analysis of variance.

Source	Sum of Squares	*df*	Mean Square	*F*-Value	*p*-Value	
Model	3455.64	14	246.83	432.32	<0.0001	Significant
A-Moisture content	50.13	1	50.13	87.8	<0.0001	
B-Temp.	36.45	1	36.45	63.85	<0.0001	
C-Liquid:solid ratio	3352.08	1	3352.08	5871.12	<0.0001	
D-Time	4.98	1	4.98	8.73	0.0098	
AB	0.0197	1	0.0197	0.0345	0.8551	
AC	0.0093	1	0.0093	0.0163	0.9	
AD	0.326	1	0.326	0.571	0.4616	
BC	0.0009	1	0.0009	0.0016	0.9685	
BD	1.02	1	1.02	1.78	0.2023	
CD	0.0456	1	0.0456	0.0798	0.7814	
A^2^	9.73	1	9.73	17.05	0.0009	
B^2^	0.9546	1	0.9546	1.67	0.2156	
C^2^	0.0669	1	0.0669	0.1172	0.7368	
D^2^	1.36	1	1.36	2.38	0.144	
Residual	8.56	15	0.5709			
Lack-of-fit	7.44	10	0.7439	3.31	0.0995	Not significant
Pure error	1.12	5	0.225			
Cor total	3464.2	29				

**Table 5:** Efficiency of multiple use of AB-8 resins.

Macroporous Resin	Use Time	Adsorption Capacity	Recovery Rate
AB-8	1	100%	108.82 ± 0.72%
	2	100%	97.34 ± 0.51%
	3	100%	92.16 ± 0.56%
	4	100%	89.91 ± 0.83%

**Table 6:** Efficiency of multiple use of deep eutectic solvent (DES).

DES	Use Time	Extraction Yield (mg/g)
DES#1	1	49.32 ± 6.12
	2	49.54 ± 3.71
	3	49.08 ± 3.12

## Data Availability

The original contributions presented in the study are included in the article, further inquiries can be directed to the corresponding author.

## Supplementary Materials

The following supporting information can be downloaded at: https://www.mdpi.com/article/10.3390/molecules31061057/s1, Table S1: Standard curves of four aescins.

## Abbreviations

The following abbreviations are used in this manuscript:

DESs	Deep Eutectic Solvents
HBDs	Hydrogen Bond Donors
HBAs	Hydrogen Bond Acceptors
BV	Bed Volume
HPLC	High Performance Liquid Chromatography
UAE	Ultrasound-Assisted Extraction
CCD	Central Composite Design
RSM	Response Surface Methodology
ANOVA	Analysis of Variance
