# Supplementary material for: Efficient Ultrasound-Assisted Extraction of Four Major Aescins from Aesculi Semen Seeds Using Deep Eutectic Solvents
Source: Molecules. 2026 Mar 23;31(6):1057. doi: 10.3390/molecules31061057 (PMC13028780; doi:10.3390/molecules31061057)
Supplement: Supplementary file 1 [file molecules-31-01057-s001.zip › molecules-4181253-supplementary.pdf]

Table S1. Standard curves of four aescins.

	Mass-concentration（mg/mL）	Regression equation	R^2
Aescin A	0.05–1	y = 5891161 x − 87892.6	0.9908
Aescin B	0.05–1	y = 5795643 x − 80110	0.9961
Aescin C	0.05–1	y = 4725139.5 x − 43828.9	0.9966
Aescin D	0.05–1	y = 6036932 x − 61564.2	0.9967
